# Living with the Past: Nutritional Stress in Juvenile Males Has Immediate Effects on their Plumage Ornaments and on Adult Attractiveness in Zebra Finches

**DOI:** 10.1371/journal.pone.0000901

**Published:** 2007-09-19

**Authors:** Marc Naguib, Andrea Nemitz

**Affiliations:** Faculty of Biology, Animal Behavior, University of Bielefeld, Bielefeld, Germany; University of Bristol, United Kingdom

## Abstract

The environmental conditions individuals experience during early development are well known to have fundamental effects on a variety of fitness-relevant traits. Although it is evident that the earliest developmental stages have large effects on fitness, other developmental stages, such as the period when secondary sexual characters develop, might also exert a profound effect on fitness components. Here we show experimentally in male zebra finches, *Taeniopygia guttata*, that nutritional conditions during this later period have immediate effects on male plumage ornaments and on their attractiveness as adults. Males that had received high quality food during the second month of life, a period when secondary sexual characteristics develop, were significantly more attractive as adults in mate choice tests than siblings supplied with standard food during this period. Preferred males that had experienced better nutritional conditions had larger orange cheek patches when nutritional treatments ended than did unpreferred males. Sexual plumage ornaments of young males thus are honest indicators of nutritional conditions during this period. The mate choice tests with adult birds indicate that nutritional conditions during the period of song learning, brain and gonad development, and moult into adult plumage have persisting effects on male attractiveness. This suggests that the developmental period following nutritional dependence from the parents is just as important in affecting adult attractiveness as are much earlier developmental periods. These findings thus contribute to understanding the origin and consequences of environmentally determined fitness components.

## Introduction

An individual's fitness is affected both by genetic factors and by the environmental conditions it experiences. Those environmental factors that act during embryonic and early postembryonic development can have strong and long-lasting effects on biometry, physiology, behaviour, reproduction and survival in humans and other animals [1]–[3]. Such effects can be negative consequences resulting from poor nutritional conditions or social stress experienced during early development or adaptive responses to environmental constraints [4], [5].

Birds have been widely used in experimental studies to obtain insights into the effects of early developmental conditions on the expression and adaptive significance of various traits [3], [6]. Studies using brood size manipulations or manipulation of food availability have shown that conditions during early development have pronounced effects on biometry and physiology [7]–[9], reproductive investment and success [10]–[12], sexually selected traits and attractiveness [13]–[17] as well as survival [18], [19]. Thus, early environmental effects need to be considered as a source of variation in traits that are relevant in evolutionary processes.

Despite the wealth of studies on the effects of early developmental stress on the expression of fitness relevant traits, much less is known about the extent to which environmental conditions during later developmental periods have sustained effects on adult performance. The developmental period in which secondary sexual characters develop is of particular interest, as these characters can reflect an individual's ability to acquire resources and to cope with environmental constraints when parental care is ending. In zebra finches, *Taeniopygia guttata* (Vieillot, 1817), this is the second month of life: the period of moult into adult plumage [20] which coincides with the period of gonadal development [21], [22] and song learning [23], [24]. Effects of nutrition on plumage development are well studied, showing that secondary sexual plumage characters are indicators of an individual's condition during moult [25], [26]. Since in zebra finches the moult into adult plumage coincides with the development of many other traits, plumage ornaments in young males could indicate a range of other physiological and behavioural traits that develop during this period.

In order to test the hypothesis that nutritional conditions during the second month of life affect subsequent sexual attractiveness, we used zebra finches that had experienced different nutritional conditions during this period in subsequent mate choice tests. Here, females were allowed to choose between sibling males that had been raised under identical conditions until day 35 post hatching but then had received different nutritional treatments from day 35 to day 60. Mate choice tests were conducted when males were about six months old. We also tested whether nutritional conditions during this period have immediate effects on sexually selected plumage ornaments.

## Results

**Figure 1:**
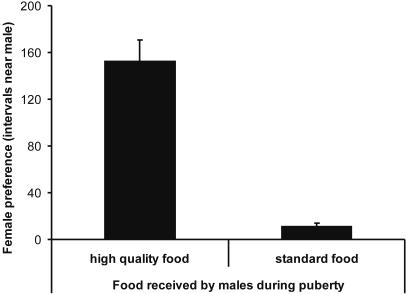
Number of 10 s intervals (means±SE) spent by females perching near the males that had received different nutritional conditions from day 35 to day 60.

**Figure 2:**
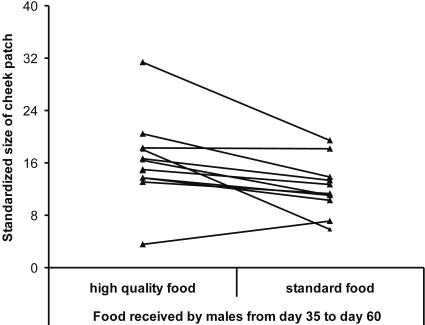
Differences in size of the orange cheek patch at day 60 of paired males as used in mate choice tests at 6 month of age.

**Figure 3:**
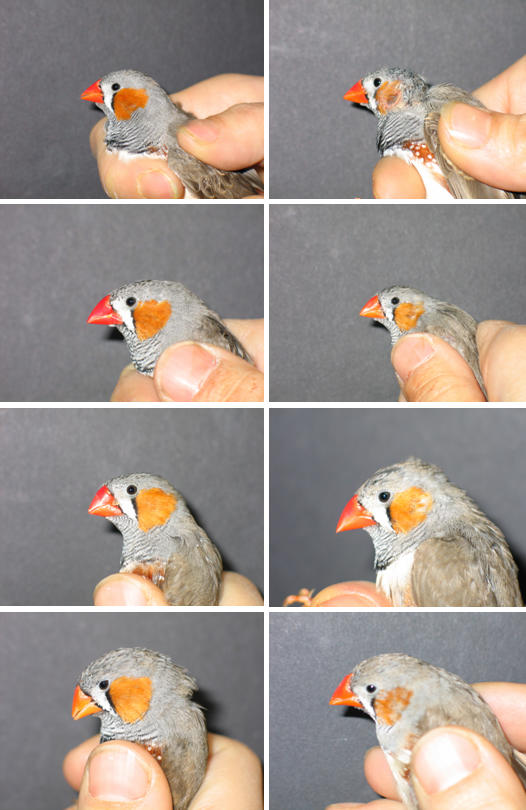
Pictures taken at day 60 from sibling juvenile males (in rows) that had experienced different nutritional conditions (left: high quality food; right: standard food) from day 35 until day 60, i.e. immediately after nutritional dependence from the parents.

The female choice tests revealed that females spent significantly more time with males that had received high quality food during the second month of life than with their counterparts that received standard food during this period (*t*_17_ = 7.72, *p*<0,0001, paired *t*-test; Fig. 1). Overall, 17 out of 18 females showed a preference for males from the better nutritional conditions and associated for on average more than 90% of the time with these males compared to the males from poorer nutritional conditions. Preferred males had significantly larger cheek patches on day 60 than did non-preferred males (*Z* = −2.20, *p* = 0.028, *n* = 12, Wilcoxon signed-ranks matched-pairs test). Among the males used in the mate choice tests, males that had received better nutritional conditions had larger cheek patches at day 60 than those that had received standard food (*Z* = −2.43, *p* = 0.015, *n* = 12; Wilcoxon signed-ranks matched-pairs test; Fig. 2, Fig. 3). Nutritional treatment had no effect on biometric traits, such as tarsus length, body mass and wing length at day 60 (all *F*_46,25_<1.02, all *p*>0.49; General linear model).

## Discussion

Female zebra finches showed a clear preference for males that had experienced better nutritional conditions during the second month of life. Previous studies showed that environmental conditions during much earlier developmental periods in life, i.e. the pre- and early post hatching period, strongly affect subsequent adult traits [1], [27] such as male attractiveness [14]. Our findings expand on these previous studies by showing that the period during which secondary sexual characters develop also has a significant effect on attractiveness even several months later.

In zebra finches the second month of life is a period of major behavioural, morphological and physiological changes. During this month the neuronal song system in the brain is still developing [23] and the gonads show a major increase in growth [21]. This is also when song learning [28], moult into secondary adult plumage and the transition of beak colour from the black in juveniles to orange in adults occurs [20]. The experimental conditions males experienced during our experiments may have affected any of these or other traits, as our experiments were designed to test if nutritional conditions in general during the second month have lasting effects on adult attractiveness rather than to manipulate specific traits. In mammals including humans, traits in adults like dominance, learning capacity or other psychological traits are known to be affected by conditions during puberty [29], [30]. Even though such effects have not yet been documented for birds, they may well exist, as the suite of events in the second month of life in zebra finches are somewhat similar to those during puberty in mammals. Indeed this early period of independence might be particularly revealing with regard to a birds own behavioural abilities and its condition. Here birds have to master environmental and social constraints on their own and this is a period of high mortality [20] and thus a period of high selection pressure. Traits that develop during this period then are more revealing with respect to a male's own abilities to acquire resources rather than its parents' abilities who are a key determinate to provide resources that are required for the traits that develop earlier. Females choosing males that experienced good conditions during this developmental period then are likely to choose those males that are more competitive and possibly of higher quality. One potential cue females might have attended to are differences in male plumage ornaments. Although we did not measure these traits at the time of the experiments, the significant difference in plumage ornaments at the end of the nutritional treatments might have persisted until adulthood and affected female choice.

Regardless of the possible role of plumage traits in the choice experiments, the significant effects on plumage are interesting in themselves with respect to their signal value. The preferred males had larger orange cheek patches than did unpreferred males at day 60, i.e. immediately after the nutritional treatments ended. These males with the larger cheek patches were also the males that had experienced the better nutritional conditions. Thus, our data show that the size of the cheek patches immediately after moult into adult plumage are indicators of nutritional conditions during the preceding month of development and at that age, patches can function as honest signals of past condition [25], [31], [32]. The size of the cheek patch has been shown recently to be also affected by social experience during this developmental period [33]. The orange cheek patches are fully developed by day 60 but intensity develops further over the weeks thereafter [20]. Our subjects were kept under identical conditions after day 60 so that males that had developed smaller cheek patches until day 60 possibly did not catch up thereafter. If so, poorer nutritional conditions led to poorer rather than delayed plumage maturation. Even though plumage characters are known to be affected by specific nutrient and protein requirements during moult [25], [26], [34] the plumage traits of young male zebra finches appear to be indicators of a specific developmental period in which many other traits develop and survival is low [35]. The sexual plumage ornaments, at least in young males, thus could be signals representing a range of condition-dependent traits that develop during this specific period.

Taken together, our findings identify the environmental conditions at the point of nutritional independence from the parents as an important source of variation in attractiveness [1], [36]. Several traits important in reproduction and thus relevant to fitness develop at this stage and males may not be able to compensate without costs for developmental stress experienced at that time. Thus, females attending to traits reflecting this specific developmental period of a potential mate are likely to make an adaptive decision, a consideration that emphasizes the importance of environmental determination of fitness-relevant traits [37], [38].

## Materials and Methods

### Subjects and experimental rearing conditions

**Figure 4:**
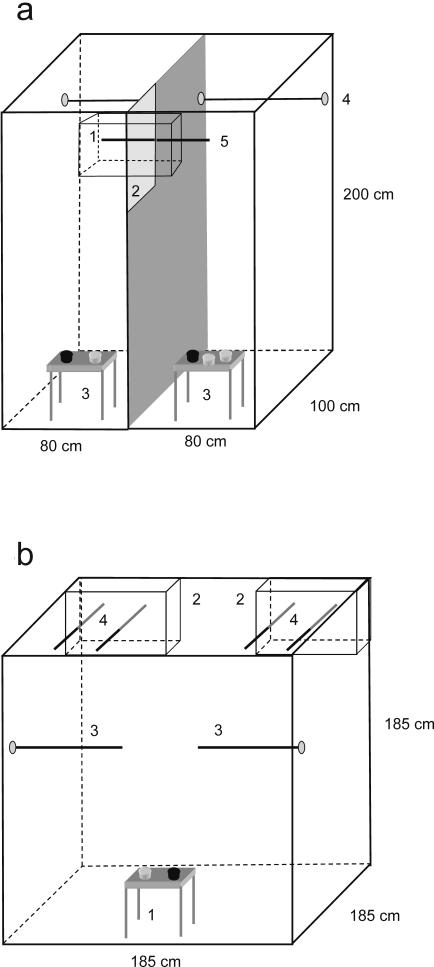
Experimental setup for periods of food manipulation and mate choice experiments (a) period of experimental food manipulation. Offspring were kept in the adjacent aviaries with one adult male as song tutor centered between the aviaries. The front of each aviary was made of mesh wire, the other parts of plywood. (1) Offspring aviaries had a perch in the rear (4) and a perch in front of the tutor (5). The separation between the two aviaries had mesh wire (2) instead of plywood between the frontal perch (5) and the front panel. Thus, offspring of the two groups could be in visual and acoustic contact to each other. Food and water was provided in dishes and differed in adjacent aviaries according to the two nutritional treatments. Overall nine of such setups were used, all located in the same experimental room. (b) mate choice experiments. Females could move freely in the large free-flight aviary. Two cages (2) with males that came from two adjacent aviaries (as shown in Figure 1a) were placed in the top rear corners of the aviaries. Females were provided with food and water (1) and perches in a neutral zone (3). Perches of male cages extended into the female cage (4) so that females could perch directly in front of either male (preference zone) without being able to see the other male. Males could not see each other due to a visual barrier between cages. Drawings are partly to scale. See text for further details

We conducted the experiments in 2004 on non-domesticated zebra finches [39](about F7 generation of wild caught birds) at the University of Bielefeld, Germany. For breeding, we kept 52 pairs in individual cages (83×30×40 cm) which were supplied daily with dried and germinated seeds, egg, and fresh water enriched with vitamins. Each cage was provided with an attached wooden nest box (12.5×12×14 cm) and with coconut fibres to be used as nesting material. Rooms had a temperature of 25°C and a L∶D regime of 16∶8 hours. Nests were checked daily for egg laying and for presence of hatchlings. In total, 21 pairs bred successfully and produced a total of 42 male and 34 female offspring. Breeding success was low in part due to first time breeding. When fledglings were 35 days old (the typical time of nutritional independence), they were transferred to mixed-sex groups of three to six individuals (size of aviaries 200×80×100 cm). They remained in these groups with different nutritional treatments until day 60, i.e. until secondary sexual characters were fully developed and until males had learned their song [20]. These mixed-sex groups were assigned to two different nutritional treatments. In both treatments subjects were supplied daily with a mixture of dried seeds and fresh water (plus additional vitamins three times a week). The high quality treatment group was additionally supplied with germinated seeds, egg food and boiled hens' eggs. Same sex siblings were assigned to different nutritional treatments during this period so that we could control for genetic effects and early developmental background. Overall we used nine such pairs of groups. Each pair of groups had one adult male as song tutor in a cage (45×24×31 cm) attached to front of the two aviaries (Fig. 4a). On day 60, all subjects were transferred to mixed sex groups in large aviaries where they received an intermediate diet consisting of standard diet supplemented by germinated seeds. Two weeks prior to the mate choice tests all subjects were transferred to same sex groups (aviary size 100×300×330 or 90×180×190 cm). At day 60, body mass was taken with a Pesola scale to the nearest 0.5 g. Tarsus length was measured with callipers to the nearest 0.01 mm, and wing length with a ruler (flattened wing) to the nearest 1 mm. On day 60, we also took two digital portrait pictures from the left side of the males' head using a Nikon Coolpix 990 digital camera. Pictures were taken from approximately 30 cm in front of a grey or black background (Fig. 3). Sample size of males from which we took pictures is lower than total sample size as we started taking these photos after some males were older than 60 days. Consequently we did not take any pictures of these males, so that photos were available for 12 out of the 18 pairs of pairs of males that were used in mate choice tests.

### Mate choice tests

When subjects were six months of age (194±47 days, mean±SD), i.e. fully adult, we tested males from the nutritional regimes for their attractiveness in female choice experiments in large free flight aviaries. Moult into adult plumage is typically completed before day 60. The subsequent wing moult then starts on average at day 80 and lasts for about 220 days [20]. Thus, at the time of mate choice experiments, experimental males were most likely to still partly be in the plumage that was developed during the period of nutritional manipulation. Mate choice tests were conducted under natural light conditions on a large terrace closed with mesh wire and a plexiglas roof, in an aviary (185×185×185 cm) equipped with food, water and natural branches (Fig. 4b). Inside the aviary, two cages (60×30×42 cm) were positioned in the two upper corners of one side. Each cage had two perches connected to its outside. The sides of the cages facing one another were covered so that males could not see each other. A female perching in front of one male could not see the other male. For mate choice tests we used 18 pairs of males, 15 of which consisted of brothers that had received different nutritional treatments but the same song tutor. In three trials, males from the two nutritional treatments were unrelated but had had the same song tutor. Males were transferred 20 min prior to the experiment's onset from their home aviary to the test cage. This change in social and acoustic environment is likely to have affected song rate in males, which was low during mate choice tests (10 sec intervals with song, preferred males: 2.5±1.2, unpreferred males 0.72±0.6, mean±SE). Thus, song was not considered in further analyses. The two males used in a given mate choice test were randomly assigned to one of the two cages in the test aviary and their identity was recorded after the mate choice test. Thus, during the tests the observer had no information on which male was in which cage. Eighteen females with breeding experience were selected randomly from our study population with the precondition that they were not directly related to the test males. Observations began immediately after females were released into the aviary. The observer sat silently about 2 m away centrally in front of the aviary. Behavioural observations lasted for 40 min, during which a female's location and male singing activity were recorded as point samples with 10 sec intervals.

### Data analysis

For data analysis we used a general linear model to test for effects of nutritional treatment on male biometric traits using sex and nutritional treatment nested within tutor group and genetic parents as factors. We used paired *t-*tests to analyze the female choice test. From the pictures, the size of the orange cheek patch was calculated using the ‘magic wand’ tool in Photoshop [33]. For each bird, the picture with the best side view of the bird's head was selected for analysis. The patch was measured three times and the average was used for further analysis. Repeated measures on the same patch correlated highly significantly with each other (*r*>0.97, *p*<0.00001). All analyses were made blind with respect to nutritional treatment. All statistics were conducted with SPSS 14.

## References

[1] Lindström J (1999). Early development and fitness in birds and mammals.. Trends Ecol Evol.

[2] Lummaa V, Clutton-Brock T (2002). Early development, survival and reproduction in humans.. Trends Ecol Evol.

[3] Metcalfe NB, Monaghan P (2001). Compensation for a bad start: grow now, pay later?. Trends Ecol Evol.

[4] Bateson P, Barker D, Clutton-Brock T, Deb D, D'Udine B (2004). Developmental plasticity and human health.. Nature.

[5] Wells JCK (2007). The thrifty phenotype as an adaptive maternal effect.. Biol Rev.

[6] Fisher MO, Nager RG, Monaghan P (2006). Compensatory growth impairs adult cognitive performance.. PLoS Biology.

[7] Brinkhof MG, Heeb P, Kölliker M, Richner H (1999). Immunocompetence of nestling great tits in relation to rearing environment and parentage.. Proc R Soc Lond B.

[8] Naguib M, Riebel K, Marzal A, Gil D (2004). Nestling immunocompetence and testosterone covary with brood size in a songbird.. Proc R Soc Lond B.

[9] Tinbergen JM, Boerlijst MC (1990). Nestling weight and survival in individual great tits (*Parus major*).. J Anim Ecol.

[10] Gil D, Heim C, Bulmer E, Rocha M, Puerta M (2004). Negative effects of early developmental stress on yolk testosterone levels in a passerine bird.. J Exp Biol.

[11] Naguib M, Gil D (2005). Transgenerational effects on body size caused by early developmental stress in zebra finches.. Biol Lett.

[12] Naguib M, Nemitz A, Gil D (2006). Maternal developmental stress reduces reproductive success of female offspring in zebra finches.. Proc R Soc Lond B.

[13] Buchanan KL, Spencer KA, Goldsmith AR, Catchpole CK (2003). Song as an honest signal of past developmental stress in the European starling (*Sturnus vulgaris*).. Proc R Soc Lond B.

[14] de Kogel CH, Prijs HJ (1996). Effects of brood size manipulations on sexual attractiveness of offspring in the zebra finch.. Anim Behav.

[15] Gil D, Naguib M, Riebel K, Rutstein A, Gahr M (2006). Early condition, song learning and the volume of brain song nuclei in the zebra finch (*Taeniopygia guttata*).. J Neurobiol.

[16] Nowicki S, Searcy WA, Peters S (2002). Brain development, song learning and mate choice in birds: a review and experimental test of the “nutritional stress hypothesis”.. J Comp Physiol A.

[17] Spencer KA, Wimpenny JH, Buchanan KL, Lovell PG, Goldsmith AR (2005). Developmental stress affects the attractiveness of male song and female choice in the zebra finch (*Taeniopygia guttata*).. Behav Ecol Sociobiol.

[18] de Kogel CH (1997). Long-term effects of brood size manipulation on morphological development and sex-specific mortality of offspring.. J Anim Ecol.

[19] Gustafsson L, Sutherland WJ (1988). The costs of reproduction in the collared flycatcher *Ficedula albicollis*.. Nature.

[20] Zann RA (1996). The zebra finch: a synthesis of field and laboratory studies..

[21] Sossinka R (1972). Besonderheiten in der sexuellen Entwicklung des Zebrafinken *Taeniopygia guttata castanotis* (Gould).. J Ornithol.

[22] Sossinka R (1980). Ovarian Development in an opportunistic breeder, the zebra finch *Phoephila guttata castanotis*.. J Exp Zool.

[23] Bottjer SW (2002). Neural strategies for learning during sensitive periods of development.. J Comp Physiol A.

[24] Catchpole C, Slater PJB (1995). Bird song: biological themes and variations..

[25] Cotton S, Fowler K, Pomiankowski A (2004). Do sexual ornaments demonstrate heightened condition-dependent expression as predicted by the handicap hypothesis.. Proc R Soc Lond B.

[26] Hill GE, Hill GE, McGraw KJ (2006). Environmental regulation of ornamental coloration.. Bird coloration Volume I Mechanisms and measurements.

[27] Groothuis TG, Muller W, von Engelhardt N, Carere C, Eising C (2005). Maternal hormones as a tool to adjust offspring phenotype in avian species.. Neurosci Biobehav Rev.

[28] Slater PJB, Eales LA, Clayton NS (1988). Song learning in zebra finches: progress and prospects.. Adv Stud Behav.

[29] Hodes GE, Shors TJ (2005). Distinctive stress effects on learning during puberty.. Hormones Behav.

[30] Kaltiala-Heino R, Marttunen M, Rantanen P, Pimpelä M (2003). Early puberty is associated with mental health problems in middle adolescence.. Soc Sci Med.

[31] Andersson M (1994). Sexual selection..

[32] Zahavi A (1975). Mate selection: a selection for a handicap.. J Theor Biol.

[33] Leader N, Nottebohm F (2006). Delayed plumage maturation in socially isolated juvenile zebra finches, *Taeniopygia guttata*.. Anim Behav.

[34] Jacot A, Kempenaers B (2007). Effects of nestling condition on UV plumage traits in blue tits: an experimental approach.. Behav Ecol.

[35] Zann R, Runciman D (1994). Survivorship, dispersal and sex ratios of zebra finches *Taeniopygia guttata* in southeast Australia.. Ibis.

[36] Hadfield JD, Burgess MD, Lord A, Phillimore AB, Clegg SM (2006). Direct versus indirect sexual selection: genetic basis of colour, size and recruitment in a wild bird.. Proc R Soc Lond B.

[37] Charmantier A, Sheldon BC (2006). Testing genetic models of mate choice evolution in the wild.. Trends Ecol Evol.

[38] Griffith SC, Owens IPF, Burke T (1999). Environmental determination of a sexually selected trait.. Nature.

[39] Forstmeier W, Segelbacher G, Mueller JC, Kempenaers B (2007). Genetic variation and differentiation in captive and wild zebra finches (*Taeniopygia guttata*).. Mol Ecol in press.

